# Both intermuscular fat and LVEF decline promote heart failure symptoms in cancer survivors

**DOI:** 10.1186/s40959-021-00102-1

**Published:** 2021-05-08

**Authors:** Kerryn W. Reding, Nathaniel S. O’Connell, Ralph B. D’Agostino, William Hundley, Alexander R. Lucas, Amy C. Ladd, Jennifer H. Jordan, Emily M. Heiston, Yaorong Ge, W. Gregory Hundley

**Affiliations:** 1 Biobehavioral Nursing and Health Informatics, University of Washington School of Nursing, Seattle, USA; 2 Fred Hutchinson Cancer Research Center Division of Public Health Sciences, Seattle, USA; 3 Department of Biostatistics and Data Science, Wake Forest School of Medicine, Winston-Salem, USA; 4 Virginia Military Institute, Lexington, USA; 5 Division of Cardiology, VCU Pauley Heart Center, Virginia Commonwealth University, Box 980036, Richmond, VA 23298 USA; 6 Department of Health Behavior and Policy, Virginia Commonwealth University, Richmond, USA; 7 Department of Biomedical Engineering, Virginia Commonwealth University, Richmond, USA; 8 College of Computing and Informatics, University of North Carolina at Charlotte, Charlotte, USA; 9 Wake Forest Department of Internal Medicine, Section on Cardiovascular Medicine, Winston-Salem, USA

**Keywords:** Cardio-oncology, Obesity, Heart failure, Intramuscular fat

## Abstract

**Background:**

Approximately 20% of cancer survivors treated with chemotherapy experience worsening heart failure (HF) symptoms post-cancer treatment. While research has predominantly investigated the role of cardiotoxic treatments, much less attention has focused on other risk factors, such as adiposity. However, emerging data in cancer survivors indicates that adiposity may also impact a variety of cardiovascular outcomes. Methods: In a prospective study of 62 patients diagnosed with cancer followed for 24 months from cancer diagnosis through to survivorship (post-cancer treatment), we ascertained baseline fat depots including intermuscular fat (IMF) of the erector spinae muscles; and pre- and post-cancer treatment left ventricular ejection fraction (LVEF) and HF symptoms at baseline and 24-months, respectively. Linear regression was used to model independent variables in relation to HF symptoms at 24-months.

**Results:**

Baseline IMF and LVEF change over 24-months significantly interacted to predict HF score at 24-months. The highest HF symptom score was observed for participants who experienced high IMF at baseline and a high decline in LVEF over 24-months (HF score = 11.0) versus all other categories of baseline IMF and LVEF change.

**Conclusions:**

Together IMF and LVEF decline may play an important role in the worsening of HF symptoms in cancer survivors. The finding that IMF at cancer diagnosis led to elevated HF scores post-treatment suggests that IMF may be a potential target for intervention studies.

## Background

Heart failure (HF) symptoms, such as activity limitations and reduced exercise capacity, worsen after cancer treatment in approximately 20% of chemotherapy-treated patients [1]. Thus far, research has focused on the role of cardiotoxic treatments to explain the increased HF symptomology under the assumption that predisposition to HF symptomology is predominantly due to left ventricular ejection fraction (LVEF) decline [2]. However, additional factors may play a role. Jones et al. showed that while a LVEF decline post-cancer treatment occurred in most chemotherapy-treated patients (mean decline of 4%), LVEF decline alone was insufficient to predict increases in HF symptoms [1].

Adiposity may contribute to heart failure symptoms in cancer survivors. A 2016 meta-analysis showed obese patients (BMI ≥ 30) were at increased anthracycline-related cardiotoxicity risk versus normal weight patients [3]. Moreover, investigations of specific depots of fat may improve the assessment of risk of cardiovascular outcomes in breast cancer beyond BMI [4, 5], an imprecise measure for body adiposity particularly in cancer survivors [6, 7]. Previously, our group investigated fat depots in relation to reduced exercise capacity in cancer survivors, a component of HF symptomology. We observed in a pilot of 14 cancer survivors an association between fat accumulated within the muscle (intermuscular fat [IMF]) and reduced exercise capacity, which persisted after controlling for LVEF decline [8]. Due to the biological plausibility that both LVEF decline and specific fat depots could contribute to HF symptomology, we sought to investigate the relationship between change in patient HF score with change in EF over 24 months and baseline IMF fat.

## Methods

This prospective study enrolled cancer patients from the Wake Forest Comprehensive Cancer Center clinics who were scheduled to receive potentially cardiotoxic cancer treatment. As previously described [4], eligibility criteria for this analysis included age > 21 years; a life expectancy of > 2 years; scheduled to receive potentially cardiotoxic chemotherapy for breast cancer, lymphoma, or soft tissue sarcoma; and receipt of abdominal magnetic resonance imaging (MRI). Patients with contraindications to a cardiovascular magnetic resonance (CMR) exam (e.g., implanted electronic devices) were excluded. We followed 62 cancer patients for 24 months starting from the time of diagnosis through to survivorship (post-cancer treatment). This study was approved by the Wake Forest Health Sciences Institutional Review Board and all participants provided written, witnessed informed consent.

CMR images to assess LVEF were acquired at baseline (i.e., prior to the first cycle of chemotherapy) and at 24 months post-baseline. Specifically, images were acquired using a 1.5 Tesla Avanto (Siemens Healthcare, Erlangen, Germany) MRI scanner. LVEF measurements were obtained using previously published methods [9], that included cine bright blood steady-state free precision techniques with 160 × 120 matrix, a 42 cm field of view, an 8-mm-thick slice with a 2- mm inter-slice gap, and a 33-ms temporal resolution. The CMR cine slices were manually analyzed using QMASS (Medis, Leiden, The Netherlands) to determine LV volumes and ejection fraction. A reader blinded to the patient and visit information manually outlined the endocardium and epicardium from the end-diastolic and end-systolic phases for the baseline and 24-month visits. The end-diastolic and end-systolic volumes as well as the LVEF were calculated according to modified Simpson’s Rule Technique from manual contours [10]. MR imaging was chosen to assess LVEF due to its accuracy and prior use in U.S. National Institutes of Health-funded initiatives, such as the MESA (Multi-Ethnic Study of Atherosclerosis) [11].

Abdominal MRI images were analyzed to generate IMF of the erector spinae muscles measured at baseline (cancer diagnosis) using previously described methods [8]. Using the 1.5 Tesla Avanto MRI scanner, abdominal scans were performed according to previously published techniques [12]. Total and compartmental amounts of abdominal fat were determined from the axial slice positioned at the level of the second lumbar vertebra (L2) with a 256*256 matrix, a 5-mm-thick slice, a bandwidth of 305 Hz/pixel, and a field of view to encompass all of the abdomen. Fat depots were separated into erector spinae IMF, abdominal SQ fat, and visceral adipose tissue (VAT) using the SliceOmatic 5.0 Rev-4b2 software program (Tomovision, Montreal, Canada) [13]. Erector spinae IMF was defined as the fat between and within the erector spinae muscle. SQ fat was defined as the fat outside the muscular abdominal wall, and VAT was defined as the fat to the interior of the abdominal wall. The bimodal distribution for muscle (lower intensity) and fat (higher signal intensity) provides the ability to distinguish fat from muscle in MRI images, which was shown to enhance precision [14]. Adipose tissues were segmented and colored from other tissues, including muscle, based on pixel intensity and known divisions of tissue planes using a SliceOmatic automated algorithm. The blinded reader corrected any misidentified fat or non-fat regions using manual tools provided within the software. To calculate each of the compartmental fat deposits, the number of subpixels within each fat compartment (IMF, SQ, VAT) was multiplied by the individual pixel dimensions within the image and by the slice thickness to determine the area of fat (in cm^2^) for each compartment.

The Minnesota Living with Heart Failure questionnaire (MLHFQ) was measured at baseline and 24-months [1]. For HF score, we used a symptom subscale of 7 MLHFQ questions focused on activity limitations including 1) shortness of breath, 2) needing to rest during the day, 3) tired, fatigued, or low on energy, 4) difficulty around the house, 5) difficulty in taking stairs, 6) difficulty going places, and 7) difficulty working. Each response followed a Likert scale from 0 to 5 in terms of increasing burden. Our primary outcome, HF score, was calculated as the sum of the ordinal responses for the 7-question subscale.

In statistical analysis, we dichotomized LVEF decline based on a drop > 5% from baseline to 24-months; and baseline IMF > 9 *cm*^2^ (corresponding roughly to the median for both). Missing data was scarce and included: IMF at baseline (*n* = 2); HF scores at 24-months (*n* = 4). Random Forest Imputation was utilized to impute these missing values. There were no missing data for LVEF. We used linear regression to test our hypothesis by modeling HF score continuously (at 24-months), while adjusting for baseline HF. Independent variables investigated in this model were change in LVEF and baseline depots of fat, namely IMF, VAT, and SQ fat.

## Results

At cancer diagnosis, participants were a mean age of 53.4 years (SD: 15.2), a mean weight of 86.1 kg (SD: 17.9). As shown in Table 1, nearly one-third of our sample were men and three-quarters were Caucasian; the mean BMI was 30.0 kg/m^2^ (SD: 5.8). Few (6.5%) had coronary artery disease, 32.3% were former or current smokers, 35.5% had hypertension, and 16.1% had Type II diabetes. Diuretics and ACE Inhibitors were the most common anti-hypertensive drugs (with 19.4 and 14.5%, respectively, currently receiving those medications); and nearly one-third were taking lipid-lowering drugs. With respect to cancer, 37.1% were diagnosed with breast cancer, 53.2% with lymphoma, and 9.7% with sarcoma; a majority received anthracyclines (67.7%). This sample of cancer survivors experienced a mean decline in LVEF of 5.1% (SD: 8.2) over 24-months.

**Table 1:** Characteristics of the study population

	Overall
	(***N*** = 62)
**Gender**	
Female	41 (66.1%)
Male	20 (32.3%)
Missing	1 (1.6%)
**Race**	
White	48 (77.4%)
Black	12 (19.4%)
Other	1 (1.6%)
Missing	1 (1.6%)
**Cancer Type**	
Breast	23 (37.1%)
Lymphoma	33 (53.2%)
Sarcoma	6 (9.7%)
**Coronary Artery Disease**	
No	58 (93.5%)
Yes	4 (6.5%)
**Smoking History**	
No	42 (67.7%)
Yes	20 (32.3%)
**Diabetes**	
No	52 (83.9%)
Yes	10 (16.1%)
**Hypertension**	
No	40 (64.5%)
Yes	22 (35.5%)
**Anthracycline**	
No	20 (32.3%)
Yes	42 (67.7%)
**Diuretic**	
No	50 (80.6%)
Yes	12 (19.4%)
**Beta Blocker**	
No	58 (93.5%)
Yes	4 (6.5%)
**ACE Inhibitor**	
No	53 (85.5%)
Yes	9 (14.5%)
**Angiotensin Receptor Blocker**	
No	58 (93.5%)
Yes	4 (6.5%)
**Calcium Channel Blocker**	
No	57 (91.9%)
Yes	5 (8.1%)
**Lipid Lowering Drugs**	
No	42 (67.7%)
Yes	20 (32.3%)
**Baseline IMF**, cm^2^	
Mean (SD)	9.13 (4.08)
**Baseline BMI**, kg/m^2^	
Mean (SD)	30.0 (5.76)
**Change in EF from Baseline**, %	
Mean (SD)	−5.12 (8.25)
**Baseline Systolic BP**, mmHg	
Mean (SD)	119 (17.5)
**Baseline Diastolic BP**, mmHg	
Mean (SD)	69.8 (13.5)

**Fig. 1:**
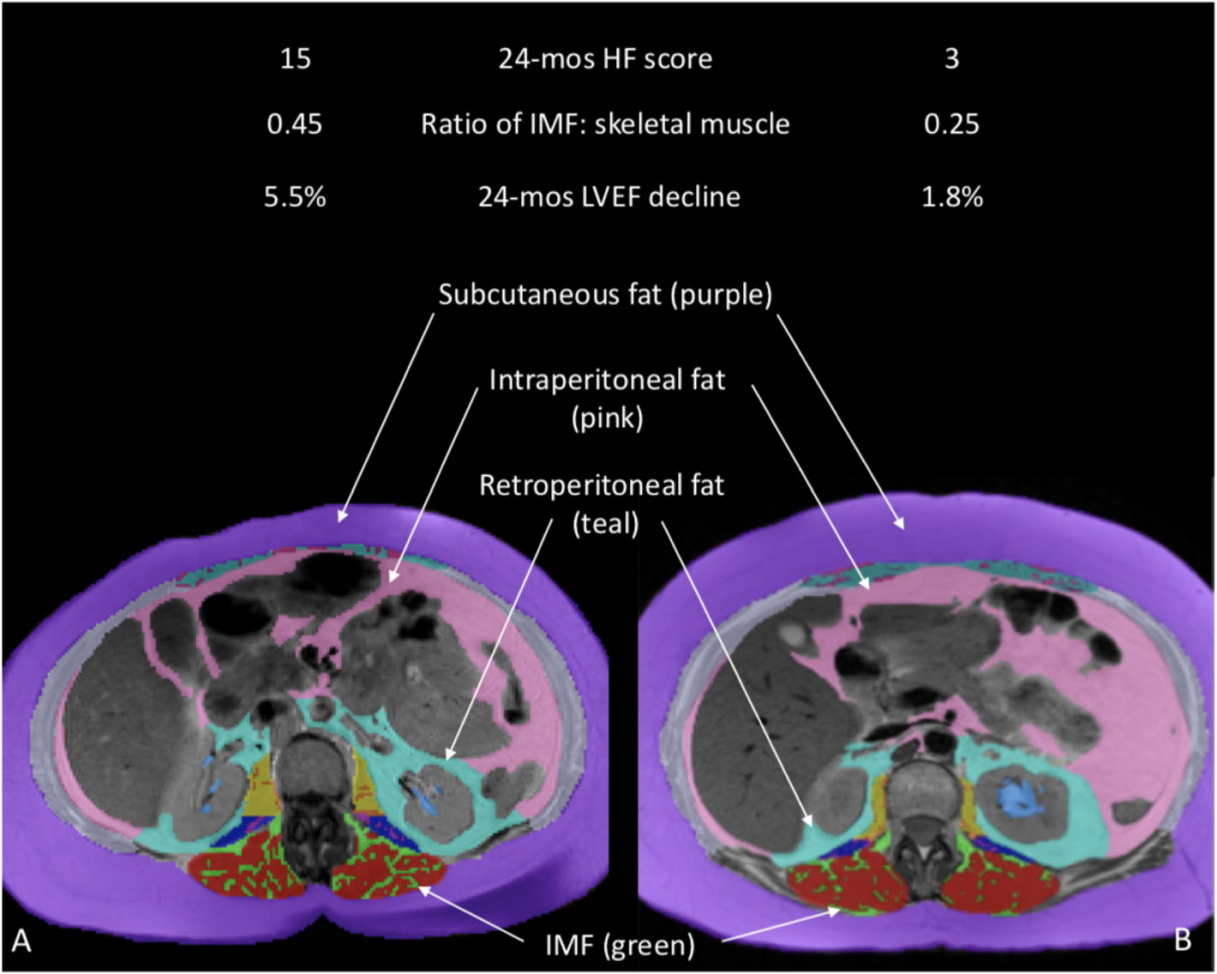
IMF differences in two cancer survivors with differing post-treatment HF symptomology. Axial images of the abdomen at vertebra L2 depicting fat depots, including intermuscular fat (IMF, shown in green) of the erector spinae muscles, in cancer survivors with high (**a**) versus low (**b**) post-treatment HF scores

Elevated IMF and LVEF decline together contributed to high HF symptomology post-cancer treatment in cancer survivors, adjusted for baseline HF score. We observed the worst 24-month HF symptom score (11.0; 95% CI: 8.6–13.4) in cancer survivors who experienced high LVEF decline and high baseline IMF, which was statistically different from those with high LVEF decline and low IMF (HF symptom score = 5.8; *p*-value = 0.01), low LVEF decline and high IMF (HF symptom score = 4.1; *p*-value = 0.001), and low LVEF decline and low IMF (HF symptom score = 3.7; *p*-value< 0.001; Fig. 1). Neither VAT nor SQ fat were associated with a change in HF 1score.

## Discussion

These data suggest that baseline adiposity within skeletal muscles when taken in conjunction with the extent of LVEF decline is an important factor in predicting HF symptomology post-cancer treatment. A difference of nearly 7 points on the 7-question MLHFQ subscale between high LVEF-high IMF and all other categories indicates a nearly 1-point drop across all HF symptoms assessed.

As many of the HF symptoms investigated relate to activity limitations, there exists strong overlap with the symptom of reduced exercise capacity. The mechanisms underlying a link between LVEF decline, IMF, and these HF symptoms may thus be understood through use of the Fick equation, which states that exercise capacity is a function of cardiac output and peripheral factors measured as the arteriovenous oxygen (AVO_2_) difference [15]. With respect to cardiac output, LVEF decline has clear implications. Simultaneously, IMF was found to be the predominant contributor to peripheral factors impairing exercise capacity in cancer survivors [16]. A primary mechanism for this is thought to be through IMF’s role in reduced skeletal muscle extraction from the vasculature [16].

The interaction observed between LVEF decline and IMF requires interpretation in the context of cancer patients experiencing accelerated damage to the heart. In this setting, patients with high baseline IMF – which may generate pro-inflammatory cytokines leading to systemic inflammation [17] – could experience greater detrimental impacts to the heart due to the inflammatory milieu at the time of cardiotoxic treatment. The finding that IMF at the time of cancer diagnosis served as a risk factor may indicate its potential as an intervenable target in this population prior to or during the receipt of cardiotoxic treatment.

## Conclusions

Our findings add to the growing literature that CVD risk factors, in particular adiposity, may predispose cancer patients to cardiotoxic effects [3, 5], by demonstrating that a particular fat depot is associated with HF symptomology. While these findings require replication prior to planning an intervention, this study raises IMF as a potential target for interventions to reduce the incidence of worsening HF symptoms in cancer survivors. Future studies of cancer survivors need to investigate excess adiposity in relation to HF symptoms, as well as lifestyle habits such as diet and physical activity in relation to excess adiposity, in order to inform interventions addressing HF symptoms.

## Data Availability

The datasets analyzed during the current study are available from the corresponding author on reasonable request.

## Abbreviations

BMI: Body mass index
Cm: Centimeter
CMR: Cardiovascular magnetic resonance
HF: Heart failure
IMF: Intermuscular fat
LVEF: Left ventricular ejection fraction
MESA: Multi-Ethnic Study of Atherosclerosis
MLHFQ: Minnesota Living with Heart Failure questionnaire
MRI: Magnetic resonance imaging
SD: Standard deviation
SQ: Subcutaneous
VAT: Visceral adipose tissue
